# Increasing character strength knowledge, interest, and skill: preliminary evidence for a collaborative and multimethod assessment procedure

**DOI:** 10.3389/fpsyg.2023.1179052

**Published:** 2023-07-27

**Authors:** Jeffrey Klibert, Michaela Simpson, Brandon Weiss, C. Thresa Yancey, Calla Pritulsky, Amy Luna, Hayley Houseman, Hani Samawi

**Affiliations:** ^1^Department of Psychology, Jing-Ping Hsu College of Public Health, Georgia Southern University, Statesboro, GA, United States; ^2^Department of Psychiatry, Mount Sinai Hospital, New York, NY, United States

**Keywords:** positive psychology, collaborative multimethod assessment, character strength, card sorting method, qualitative assessment

## Abstract

**Introduction:**

The study’s objective was to evaluate whether a qualitative, collaborative, and multimethod assessment protocol increased reports of character strength interest, knowledge, and perceived skills.

**Methods:**

Thirty-two participants completed three phases of data collection. Participants were first screened for well-being, which was used as an auxiliary covariate to order participants into experimental conditions. Selected participants were randomly assigned to a control or collaborative and multimethod assessment (card sort × qualitative interview) condition. Participants completed pre- and post-measures of strength interest, knowledge, and perceived skill. In the final phase, second phase participants were invited to report on strength-related outcomes 24 h post-administration using an online survey.

**Results:**

A series of 2 (Assessment Condition) × 3 (Time) mixed ANOVAs were analyzed. Results revealed a significant assessment condition by time interaction for strength knowledge and perceived skill. Participants in the collaborative and multimethod assessment condition reported higher strength knowledge and perceived skills compared to control participants. These effects were maintained for 24 h.

**Conclusion:**

The findings offer preliminary yet sizable support for using collaborative and multimethod assessment procedures to increase strength knowledge and perceived skill. Because of the qualitative, collaborative, and individualized nature of our assessment protocol, the findings offer a low-cost and contextually bound pathway to increase strength-based outcomes.

## Introduction

Know and invest in your character strengths, and you will build more opportunities to live within your best life. This maxim is a fundamental pillar guiding the theory and practice of positive psychotherapy (Seligman and Csikszentmihalyi, 2000). From an empirical perspective, research consistently highlights the benefits of recognizing and capitalizing on character strengths to promote a wide range of positive psychological outcomes, like happiness (e.g., Schutte and Malouff, 2019). Despite the inherent potential for character strengths to stimulate thriving, questions remain regarding the best method of introducing people to strength-based concepts. Specifically, researchers call for developing and rigorously evaluating strength-based assessment procedures designed to increase important character strength outcomes (Owens et al., 2015). In response, the impetus of the current study was to evaluate whether a collaborative and multimethod procedure increases reports of strength knowledge, interest, and perceived skill.

### Character strengths

Although there are 12 distinct criteria guiding the classification of character strength (Peterson and Park, 2009), researchers largely define it as a collection of positive attributes an individual possesses, celebrates, and capitalizes on to achieve a sense of well-being or fulfillment (Peterson and Seligman, 2004). These traits are often the focal point by which individuals describe their identities and serve as energizing and intrinsically motivating forces to identity development (Linley, 2008). The significance of character strength in promoting positive outcomes across different life domains is well established. Notably, character strengths are influential in boosting flourishing, decreasing symptoms of psychopathology (Schutte and Malouff, 2019), increasing employee task performance (Pang and Ruch, 2019), and elevating levels of engagement and hope (Madden et al., 2011).

Despite these advantages, there are significant points of disengagement with character strengths regarding how they are discussed and fostered across different social, cultural, community, organization, and academic institutions (Niemiec, 2018). Research estimates two-thirds of people are unaware of their character strengths (Linley, 2008). Moreover, people attend to cultural mores minimizing the function and utility of character strength. For instance, people find more value in the idea of “fixing weaknesses” vs. “building on strength” to achieve success (Buckingham, 2007). Finally, even when people have opportunities to invest in character strengths, they can temporarily forget or eschew the psychological benefits of leaning on their individual strengths in times of adversity and challenge (Smith and Barros-Gomes, 2015).

In acknowledgement of institutional disengagement, most practitioners initiate strength-based interventions with a robust discussion of the purpose, function, and psychological benefits of character strength (Rashid and Seligman, 2018). This process includes using valid and reliable measures to pinpoint signature (top-rated) and phasic (lower-level) strengths (Niemiec, 2018). Currently, there are a host of psychometrically sound measures of strength, including the Strength Finder (Buckingham and Clifton, 2001), Adult Needs and Strength Assessment (Nelson and Johnston, 2008), Realize 2 (Linley, 2008), Character Strengths Rating Form (Ruch et al., 2014), and Encouragement Character Strength Scale (Wong et al., 2019). However, the Values in Action-Inventory of Strengths (*VIA*-IS; Peterson and Seligman, 2004) is the gold standard. The *VIA*-IS measures 24 durable and universal character strengths through a standardized, quantitative assessment procedure (McGrath, 2014; see http://www.viacharacter.org/www/Character-Strengths-Survey). The *VIA*-IS provides respondents with a wealth of practically valuable insights through multiple reports about their strength profile. Specifically, reports outline a comprehensive description of top-and lower-level strengths, over/under use of strengths, tips for boosting lower-level strengths, and benefits of employing different character strength combinations in the pursuit of well-being. Although the effects of administering the *VIA*-IS on strength-concordant outcomes (i.e., enhanced knowledge, interest, skill) is under-researched, a handful of studies offer evidence for the effectiveness of such procedures, particularly in elevating reports of strength knowledge and use (e.g., Forest et al., 2012).

The *VIA*-SI is a valuable instrument, offering a thorough evaluation of character strengths. Notably, although the factor structure of the *VIA*-SI is consistently refined and improved (Ng et al., 2017), the 24 strength factors underlying the latent construct demonstrate high levels of internal consistency, test–retest reliability, and convergent and predictive validity (Seligman et al., 2004; Park, 2021). The online administration of *VIA*-SI yields a wide variety of generalized strength profiles based on unique elevations of strength scores. Typically, these reports contain detailed descriptions based on the most common expressions of individual strength scores (see http://www.viacharacter.org/www/Character-Strengths-Survey). Yet, due to the standardized nature of the assessment procedures and generalized nature of the feedback offered, the *VIA*-SI does have inherent limitations potentially impacting how individuals grow within different strength-concordant outcomes. Specifically, over-reliance on frequency data (elevations) and descriptions of common/generalized characterizations to frame strength profiles minimizes the idiosyncratic and contextualized expressions of strengths (Laher and Cockcroft, 2017; Klibert and Allen, 2019). To remedy these limitations, researchers recommend creating more individualized strength-based assessment procedures, specifically those to help individuals identify relevant strengths and discuss how strengths are expressed, celebrated, and exercised within the context of different life experiences (Ruch et al., 2020).

Emerging literature highlights multiple attempts to contextualize accessing and expressing strengths through dynamic measurement procedures. In the context of Positive Psychotherapy (Rashid and Seligman, 2018), an evaluation of character strengths is a core feature of how individuals increase different strength-concordant outcomes. Measurement of strengths is largely dependent upon standardized and validated self-report scales (e.g., *VIA*-SI), but also includes contextualizing and framing strengths within the lived experience of respondents. Contextualized methods are often investigated through worksheets, where respondents explore how specific strengths are accessed, utilized, and expressed in different spaces. Insights obtained from these explorations are supplemented by others’ reports (e.g., friends, family) to reinforce strength-based knowledge and use. Despite the depth and richness generated from these worksheets, the platform used is based on standardized definitions and descriptions of strengths, risking the minimization of unique manifestations and expressions of strength for each respondent (Klibert and Allen, 2019).

In response to this concern, other researchers are exploring more qualitative methods of assessing character strengths. For instance, a handful of open-ended strength-based questions included in larger intake processes about mental health (Flückiger et al., 2009) or independently driven explorations through semi-structured interviews (Scheel et al., 2013) are being administered to contextualize how respondents define, access, use, and extend strengths. Interview questions invite respondents to evaluate the presence of strengths in important life experiences, the limitations of strengths across different settings, how strengths are framed, how strengths support identity growth, the benefits of using strengths in daily life, and how using strengths increases a personal sense of meaningfulness and purpose in life. Other researchers employ content analysis on personal notes and letters to evaluate specific character strengths. Notably, Wong et al. (2019) developed a coding manual to assess unique descriptions and expressions in strengths of encouragement.

Qualitative procedures employ an open-ended, broad, and unassuming scope to evaluate individualized expressions of strength (Klibert and Allen, 2019). Respondents frame their experiences in a contextual format consistent with their values, identities, and social context (Hays, 2008; Goodwin et al., 2018). From these data, a respondent’s preferential language, adaptation processes, and unique roles in the expression of strength are illuminated (Wright and Lopez, 2002; Owens et al., 2015). In turn, these highly individualized expressions offer valuable pathways allowing respondents to meaningfully digest and use insights to promote greater levels of strength-concordant outcomes (Klibert and Allen, 2019). In total, qualitative procedures offer a respondent-led, rich, and personally meaningful definition and description of strengths. However, these procedures are not well organized into a larger system of assessment and lack empirical support regarding their effects on strength-concordant outcomes.

Collaborative and multimethod assessment (CMA), like Therapeutic Assessment (Finn and Tonsager, 1997), is an approach potentially addressing limitations in how character strengths are currently identified and discussed. The major thrust of CMAs is to maximize the interventional qualities of assessment procedures; they are widely considered to promote self-exploration, increase positive experiences, improve psychological functioning, and prepare individuals for further intervention (Finn, 2007). Regarding design, CMA approaches are often characterized by collaborative and highly individualized procedures (De Saeger et al., 2014). Typically, CMA combines standardized assessment and semi-structured interviewing procedures to help respondents find meaning and invest in their test feedback, though CMAs are not tied to any specific form of psychological assessment process (Finn, 2007). Within CMA, assessment findings serve as the starting point for cooperative action with respondents, forming pathways toward self-exploration and self-verification (Yalch et al., 2021). Assessors play an active role in the self-exploratory process; they use an individualized and non-judgmental approach, helping respondents translate assessment findings into the idiographic context of their lived experience (De Saeger et al., 2014; Aschieri, 2016). For instance, assessors never assume they fully understand the meaning behind a respondent’s scores and answers. Semi-structured interviewing opens the door for client involvement in exploring how scores/responses intersect with their identity, level of functioning, and socio-cultural context (Aschieri, 2016; Smith, 2016), thereby supporting more meaningful emotional and behavioral growth. Overall, CMA is a dynamic model of assessment, expanding upon the limitations of traditional methods of information-gathering assessment and fostering greater opportunities for self-discovery and growth through collaborative reviews of respondents’ scores and answers.

In terms of research, most evaluations remark on if and how CMA procedures affect a respondent’s readiness for treatment and their experience of psychological symptoms (Poston and Hanson, 2010; Aschieri et al., 2015). However, research highlights CMA as a useful tool promoting self-enhancing outcomes (Durosini and Aschieri, 2021). Importantly, meta-analytic studies indicate CMA is effective in promoting a diverse range of therapeutic benefits, including greater levels of self-efficacy, self-esteem, empathy, and life satisfaction (Hanson and Poston, 2011; Durosini and Aschieri, 2021). Moreover, qualitative reports indicate respondents experience increased interest in test scores, knowledge about emotional and behavioral functioning, self-awareness regarding the formation and expression of their identities, and other strength-based attributes, like empowerment and validation (De Saeger et al., 2016; Smith and Egan, 2017). Likewise, experimental research underscores the advantages of employing CMA procedures regarding different strength-based outcomes; CMA increases self-knowledge and understanding, perceptions of self-worth and self-progress, positive expectations for change, interpersonal bonds and engagement with others, hope, and satisfaction with service (Newman and Greenway, 1997; Allen et al., 2003; De Saeger et al., 2014).

Although research clearly highlights CMA as a promotional factor to self-enhancement outcomes (Durosini and Aschieri, 2021), few, if any, CMA procedures explore, increase, and extend strength-based concepts underlying the Three-Pillars (i.e., positive experiences/emotions, character strengths, and engagement with positive institutions) of Positive Psychology model (Seligman and Csikszentmihalyi, 2000). For instance, there is a dearth of research exploring how CMA procedures help respondents find meaning and invest in feedback stemming from core assessments in positive psychology (e.g., *VIA*-SI).

### Current study

Drawing on the need to create more individualized strength-based assessment procedures (Ruch et al., 2020), we developed a brief assessment protocol consistent with the tenets of CMA (Finn, 2007). The purpose of the current study was to determine whether our brief assessment protocol increases self-reports of strength-based outcomes, including strength interest, knowledge, and perceived skills. Based on CMA-applicable theory and empirical work, we hypothesized individuals participating in our brief assessment protocol would report increases in strength-based knowledge, interest, and perceived skill ratings compared to control group participants. By examining this line of inquiry, we hope to provide an empirical foundation for assessors to employ different CMA protocols supporting character strength growth in a diverse range of respondents.

## Materials and methods

### Participants

Undergraduate students at a large southeastern university in the United States were recruited through an online data recruitment system. Most traditional undergraduates in the United States are between the ages of 18 and 25. Developmentally, these students are classified as emerging adults and are in the process of forming and solidifying their personal identities (Arnett, 2015). Considering strength-based development clearly coincides with identity growth (Walker et al., 2016; Palmer et al., 2023), emerging adults appear to be a well-suited group to benefit from strength assessment interventions.

Participants were able to complete multiple (up to three) phases of the study. In Phase 1, 411 participants with an average age of 21.96 (*SD* = 5.06) years completed initial procedures. In Phase 2, 41 students with an average age of 21.34 (*SD* = 2.26) years completed follow-up procedures. In Phase 3, 32 participants with an average age of 21.23 (*SD* = 2.05) years completed the final portion of the study. Socio-demographic frequency data for each sample are reported in Table 1. Participants received course credit and/or extra credit for participating in each phase of the study.

**Table 1:** Socio-demographic characteristics of participants.

	Phase 1	Phase 2	Phase 3
Demographic variable	*n = 411*	*n = 41*	*n = 32*
Gender			
Cisgender man	58 (15.8%)	7 (17.1%)	4 (12.5%)
Cisgender woman	309 (84%)	34 (82.9%)	28 (87.5%)
Other	1 (0.3%)	0 (0%)	0 (0%)
Race/Ethnicity			
White/Caucasian	210 (57.1%)	21 (51.2%)	17 (53.1%)
African American/Black	116 (31.5%)	13 (31.7%)	10 (31.3%)
Asian/Asian American	4 (1.1%)	0 (0%)	0 (0%)
Mexican American/Latino/a	18 (4.9%)	5 (12.2%)	4 (12.5%)
American Indian/Native American	3 (0.8%)	0 (0%)	0 (0%)
Multiracial	15 (4.1%)	2 (4.9%)	1 (3.1%)
Other	2 (0.5%)	0 (0%)	0 (0%)
SES status			
Poor/Impoverished	27 (7.3%)	3 (7.3%)	3 (9.4%)
Some financial resources	224 (60.9%)	24 (58.5%)	18 (56.3%)
Substantial financial resources	114 (31.0%)	14 (34.1%)	11 (34.4%)
Affluent/Rich	3 (0.8%)	0 (0%)	0 (0%)
Population of hometown residence^*^			
Under 10,000	80 (21.7%)	12 (29.3%)	9 (28.1%)
Between 10,001 and 20,000	57 (15.5%)	4 (9.8%)	3 (9.4%)
Between 20,001 and 30,000	47 (12.8%)	4 (9.8%)	4 (12.5%)
Between 30,001 and 40,000	33 (9.0%)	2 (4.9%)	2 (6.3%)
Between 40,001 and 50,000	14 (3.8%)	6 (14.6%)	4 (12.5%)
Between 50,001 and 60,000	18 (4.9%)	1 (2.4%)	1 (3.1%)
Between 60,001 and 70,000	11 (3.0%)	0 (0%)	0 (0%)
Between 70,001 and 80,000	15 (4.1%)	2 (4.9%)	2 (6.3%)
Between 80,001 and 90,000	6 (1.6%)	0 (0%)	0 (0%)
Between 90,001 and 100,000	11 (3.0%)	2 (4.9%)	1 (3.1%)
Between 100,001 and 500,000	50 (13.6%)	6 (14.6%)	5 (15.6%)
Between 500,001 and 1,000,000	11 (3.0%)	0 (0%)	0 (0%)
Over 1,000,000	14 (3.8%)	1 (2.4%)	0 (0%)

^*^One person did not report population of hometown residence in all three phases of data collection.

### Procedure

**Figure 1:**
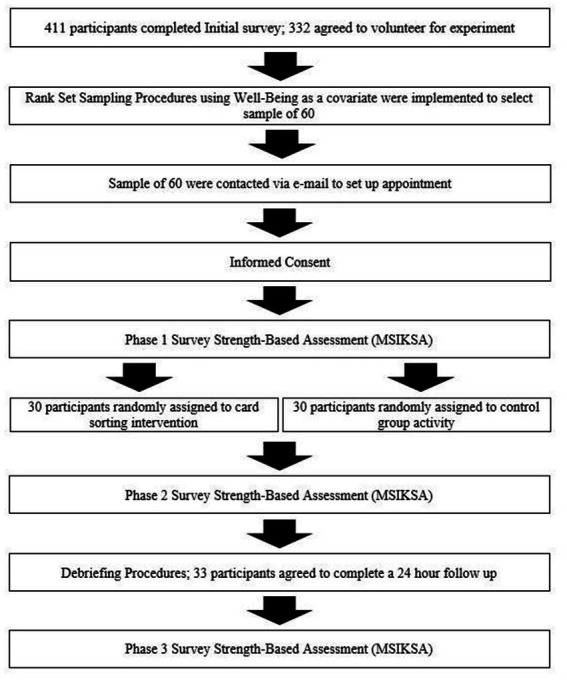
A flow chart of the study’s procedures.

A flow chart of the study procedures is presented in Figure 1. Initially, participants (*N* = 411) were recruited, via an online platform, to complete an online survey, providing information pertaining to socio-demographics characteristics and well-being (Ryff and Keyes, 1995). Interested students evaluated multiple studies for potential participation via SONA,^1^ an online portal commonly used to recruit undergraduate students for involvement in diverse research activities. Those interested in our study read a brief abstract about the nature of the study and reviewed the risks and benefits via an informed consent sheet. If students provided informed consent, they were transported to a Qualtrics survey where they anonymously completed the self-report surveys. At the end of Qualtrics survey, Phase 1 participants indicated their willingness to take part in the in-person, experimental portion of the study (Phase 2). Of the initial 411 participants, 202 (49.12%) volunteered.

Considering how undergraduate students vary in their knowledge and perceptions of psychological strengths, we choose to employ Rank Set Sampling (RSS; McIntyre, 1952) with an auxiliary covariate procedure to assign participants to groups. Previous research indicates that RSS procedures improve the precision by which treatment effects are detected (Donner and Zou, 2007) and reduce the sample size needed to detect practically significant treatment effects (Egger et al., 1985) compared to simple random sampling. In the current study, we employed a modified RSS procedure. Specifically, we used an auxiliary covariate to order participants into different groups to ensure important characteristics were evenly dispersed across control and CMA conditions, consistent with field recommendations (Jabrah et al., 2018). Well-being scores obtained from the screener survey in Phase 1 served as the auxiliary covariate. This choice seemed prudent as well-being is strongly correlated with several strength features (Karris Bachik et al., 2020). In addition, well-being scores were successfully employed as a ranked covariate in other positive psychological studies (Klibert et al., 2019).

Participants volunteering to complete Phase 2 were randomly shuffled and divided into groups based on their well-being scores. Using randomization, we selected 60 (29.7% of those interested in Phase 2) participant volunteers from Phase 1. The sample size for Phase 2 was based on an estimation of power using Minitab software. Specifically, we wanted to ensure our sample size allowed detection of small effects; therefore, we calculated sample size using a power score of 0.80 and an error probability score of 0.05. We estimated the variance of the difference between control and intervention groups using metrics generated from a previous study (Klibert et al., 2019). The resulting sample size was 60. Using these 60 participant volunteers, we compiled two 30-participant groups with comparable well-being scores. We randomly assigned each 30-participant group to a condition (control vs. CMA) via a coin toss.

Employing RSS procedures ensures equity in the auxiliary covariate, especially between individuals selected and not selected to participate in the second phase of the study. RSS generates a more representative sample with respect to the auxiliary variable, especially when compared against simple random assignment (Donner and Zou, 2007). As a result, RSS procedures increase the likelihood of comparable well-being scores for individuals selected vs. not selected for inclusion in the second portion of the study, which was confirmed by a simple ANOVA on well-being scores, *F*(1, 200) = 0.35, *p* = 0.56., *_partial_* η*p*^2^ < 0.01.

Selected participants were emailed to schedule an in-person appointment. Of those 60 participants, only 41 (68%) were able to complete Phase 2 with COVID-19 restrictions in place. At the beginning of the experiment, participants read and signed an informed consent form and completed a baseline measure of mental strength knowledge, interest, and perceived skills. Next, participants completed a control or CMA condition (see below). Participants were assigned to participate in one of these two conditions based on the pre-selected groups established through the RSS procedures. After completing the conditions, participants again completed the mental strength knowledge, interest, and perceived skills measure. At the end of the experiment, all participants were invited to participate in Phase 3 of the study. All 41 participants reported interest in completing Phase 3 of the study. Phase 3 consisted of completing one final administration of our mental strength knowledge, interest, and perceived skills measure 24 h after the administration of Phase 2 procedures. The primary purpose of the third administration of the survey was to determine whether participants completing the CMA condition sustained higher levels of strength-concordant outcomes over time. This was an important consideration as positive psychological research is heavily criticized for relying too heavily on cross-sectional studies (van Zyl et al., 2023). The researchers emailed each participant an online link to complete the final administration of our measure. A total of 32 (78%) participants completed the assessment.

### Conditions

#### Brief strength-based CMA protocol

Our brief assessment protocol is collaborative and multimethod, consistent with CMA guidelines (Finn, 2007). Although most CMA procedures employ a standardized assessment to generate test data, we implemented a qualitative procedure, a card sort, to provide greater opportunities for collaboration and individualization. Card sorting procedures were adapted from the field of career assessment, where increasing knowledge, interest, and perceived skill is a focal goal (Slaney et al., 1994; Storlie and Byrd, 2017). Consistent with common CMA practices (Smith, 2016), the card sorting portion of the protocol was followed by a brief semi-structured interview helping participants review and discuss personally meaningful findings from the card sort. Under the supervision of a licensed psychologist trained in CMA procedures, an advanced doctoral student in clinical psychology completed all administrations of the brief assessment protocol.

Participants randomly assigned to the CMA condition completed a qualitative card sorting task. Initially, participants heard the following instructions:

“I am going to invite you to participate in a card sorting task. Here is a deck of index cards. Each card is associated with the name of a mental strength that people use to help them thrive. What I’m going to ask you to do is sort them based on how ACCESSIBLE these strengths are to you, meaning how easily you’re able to use these strengths in your life. When sorting the cards, place each under one of these four categories. The categories represent different groups of accessibility. Please place each card under one of the following categories: ‘very easy to access,’ ‘easy to access,’ ‘somewhat easy to access,’ or ‘difficult to access.’ Take as much time as you need. Go ahead.”

Participants sorted 72 mental strength cards (3 × 5-in index cards). On the top of each card was a mental strength label derived from the *VIA* Classification System, which identifies 24 domains of mental strength (Peterson and Seligman, 2004). Strength domains were represented by three cards, the commonly used *VIA* label and two additional trait-based synonyms, consistent with field recommendations (Klibert and Allen, 2019). For instance, there were three cards reflecting the mental strength of *Bravery* (i.e., bravery, valor, and courage). Synonyms for each mental strength were taken from a review of Niemiec (2018). Including multiple (x3) card labels for each domain is important in capturing variations in how people define, perceive, and describe their idiographic representation of character strengths (Klibert and Allen, 2019).

After participants sorted their cards, they identified one mental strength to focus on during the next stage of the CMA protocol. To aid participants in choosing, the research assistant provided the following guidelines:

“Thank you for sorting these cards. Now, I am going to ask you to look at the cards you placed under the “Very Easy to Access” pile. When you look at these cards, please choose the one that best represents who you are. Please review these cards and let me know when you have made your choice.”

Once participants chose their card, they engaged in a brief semi-structured interview with the research assistant consisting of five open-ended questions. Specifically, participants provided a personally meaningful definition of the mental strength concept they chose (e.g., “*How do you define Kindness?*”). They also considered and provided insights into how they express their identified mental strength in everyday life (e.g., “*How do you express Bravery in your life?*”), and discussed how their identified mental strength helps them achieve success (e.g., “*How does Prudence help you accomplish your goals?*”), manage challenge (e.g., “*How do you use Humor to help you navigate challenging circumstances?*”), and increase positive emotions (e.g., “*When you are able to express Zest, how do you feel about yourself?*”). These questions were developed from the work of Scheel et al. (2013) who advocate for the use of qualitative procedures in evaluating strengths. Participants completed the entire CMA task in approximately 45 min.

#### Control task

The purpose of the control task was to simulate a similar framework for participants engaged in a card sorting task. The only difference between the control and CMA tasks was the content. Specifically, the control participants sorted cards associated with a relatively inconsequential psychological theme, familiarity with trees and plants. Participants sorted 72 index cards with the names and pictures of common trees and plants using the following instructions:

“I am going to invite you to participate in a card sorting task. Here is a deck of index cards. Each card is associated with the name and picture of a tree/plant. What I’m going to ask you to do is sort them based on how FAMILIAR these trees/plants are to you, meaning how easily you can recognize them based upon your experience. When sorting the cards, place each under one of these four categories. The categories represent different groups of familiarity. Please place each card under one of the following categories: ‘See Very Regularly/Very Familiar,’ ‘See Regularly/Moderately Familiar,’ ‘See Occasionally/Slightly Familiar,’ or ‘Rarely See/Not at All Familiar.’ Take as much time as you need. Go ahead.”

After sorting the cards, participants completed a similar process as participants in the CMA condition. Specifically, participants identified the one tree/plant with which they were most familiar. The research assistant read the following instructions:

“Thank you for sorting these cards. Now, I am going to ask you to look at the cards you place under the ‘See Very Regularly/Very Familiar’ pile. When you look at these cards, please choose the one that you are most familiar with. Please review these cards and let me know when you have made your choice.”

Once the most familiar card was identified, the research assistant engaged each participant in a five question, open-ended interview. Notably, participants discussed several unique elements about their selected card, including answering questions regarding the familiar aspects of the identified tree/plant (e.g., “*Of all the cards you evaluated, what about a Yellow Birch Tree is most familiar to you?*”), the landscape in which participants are most likely to view the identified tree/plant (e.g., “*Where are you most likely to see a Cucumber Tree?*”), participant perceptions of the purpose and/or function of the identified tree/plant (e.g., “*In your opinion, what is the purpose of a Eastern Cottonwood Tree?*”), the most striking elements of the identified tree/plant (e.g., “*When you look at an Oak Tree what aspect of it strikes you the most?*”), and descriptions of the type of climate in which the identified tree/plant is most likely to thrive (e.g., “*What type of landscape do you think a Spruce Tree would thrive in and why?*”). Like CMA participants, control participants completed the entire condition in approximately 45 min.

### Measures

#### Ryff scales for psychological well-being-short form

The Ryff scales for psychological well-being-short form (RSPWB-SF) measures different dimensions of well-being (Ryff and Keyes, 1995), including autonomy, environmental mastery, personal growth, positive relations with others, purpose in life, and self-acceptance. The 18 items are rated on a six-point scale from 1 (*Disagree Strongly*) to 6 (*Agree Strongly*). Sample items include, “*I judge myself by what I think is important, not by the values of what others think is important*” and “*I am quite good at managing the many responsibilities of my daily life*.” Total scores served as the ranked covariate in the current study and ranged from 18 to 108, with higher scores reflecting greater psychological well-being. Psychometrically, the RSPWB-SF demonstrates solid internal consistency (Klibert et al., 2019) and excellent convergent validity with different positive psychological constructs (Ryff and Keyes, 1995). In the current study, the RSPWB-SF demonstrated good internal consistency (α = 0.82).

#### Mental strength interest, knowledge, and perceived skill measure

The research team developed a brief measure of mental strength interest, knowledge, and perceived skill. The impetus behind building a measure rather than using an existing one was simple: there were no known measures to adequately assess different dimensions of strength-based functioning. In constructing the measure, items were derived from major focal points in assessing and discussing the function and purpose of mental strengths for community adults and individuals seeking mental health services (Niemiec, 2018; Klibert and Allen, 2019). The research team followed the organizational guidelines offered by Clark and Watson (2019). Notably, the team created a moderately-sized pool of items (*N* = 25). The initial set of items was evaluated for overlapping content, double-barreled content, and grammatical and sentence structural concerns by a team of applied behavioral health graduate students. Based on this review, the item pool was reduced by 11 items. The remaining items (*n* = 16) were sent to four reviewers holding expertise in the field of applied health and positive psychology. These experts rated each item based on fit with current theoretical descriptions of strength-based attributes. Specifically, expert reviewers rated the fit for each item using a 10-point scale (1 = *extremely low fit* to 10 = *extremely high fit*). Responses from each reviewer were aggregated and items with mean scores above eight were kept in the final pool of items. In total, eight items were retained and administered to the participants in Phases 2 and 3 of the current study.

Participants self-reported on the eight items regarding their familiarity with the mental strength concepts, their interest in learning more about mental strengths, and their ability to identify and use their mental strengths to enhance their quality of life in different contexts. Sample items include, “*How familiar are you with your own specific psychological or mental strengths?*,” “*How much interest do you have in learning more about your psychological or mental strengths?*,” and “*How easy is it for you to access your psychological or mental strengths to complete your goals?*” Each item was rated on a five-point scale ranging from 1 (*very unfamiliar/unimportant*) to 5 (*very familiar/important*). Score ranges varied by the identified factors: strength knowledge (2–10), strength interest (2–10), and perception of strength skills (4–20).

### Analytic plan

First, we addressed some unique features potentially threatening our ability to accurately answer the study’s main questions. Because only 41 of the 60 invited participants completed Phase 2, we conducted a series of one-way ANOVAs to determine if attrition negatively affected the internal and external validity of the study. Notably, we evaluated whether those who were invited to participate but did not complete Phase 2 differed on the rank covariate variable (well-being) when compared to participants who were invited and did complete Phase 2. We also evaluated whether attrition affected potential differences among individuals assigned to the control and CMA groups on well-being scores. In addition, we evaluated basic psychometric properties of our mental strength interest, knowledge, and perceived skill measure. Specifically, we ran an exploratory factor analysis (EFA) to determine if the measure yielded multiple factor scores consistent with our expectation. EFAs are commonly used to evaluate multidimensionality among highly complex and dynamic outcomes in psychological literature (Barendse et al., 2015). Finally, we ran a series of 2 (Assessment Condition) × 3 (Time) mixed factorial ANOVAs to address the study’s main hypotheses. Within our analysis, assessment condition (control group vs. CMA group) served as the between-participant independent variable and time (Time 1, Time 2, and Time 3) served as the within-participant independent variable. Self-reported mental strength interest, knowledge, and perceived skills served as the dependent variables. To deconstruct significant interaction effects, independent one-way ANOVAs were performed. There are several advantages of running mixed factorial designs, including the ability to examine delineated effects (interactions) and maintain higher levels of efficiency (requires fewer participants to sustain adequate power; Collins et al., 2009).

## Results

### Description of sample

Regarding the sociodemographic characteristics of the sample, there are a few patterns worth noting to better describe the population under investigation. The sample was largely comprised of emerging adult women, typical of studies sampling from undergraduate populations (Klibert et al., 2019). There was significant representation from White and Black/African American emerging adults across all three phases of the study with smaller representation from LatinX and multiethnic emerging adults. Regarding socioeconomic status, most participants reported having some or substantial resources, suggesting a moderate level of financial assets. Interestingly, a large proportion of participants reported residing in a rural or small town. For instance, in Phase 2, 68% of participants reported living in a small city/town with less than 50,000 residents. Areas containing less than 50,000 residents are considered a micro area and encompass a substantial number of rural and underserved communities (Health Resources and Services Administration, 2022).

### Preliminary findings

#### Well-being manipulation checks

Because there was significant attrition from Phase 1 to Phase 2 within the study, we evaluated whether individuals who were invited but did not participate in Phase 2 (*n* = 19) significantly differed on well-being scores (the ranked covariate) when compared to individuals who were invited and did participate in Phase 2 (*n* = 41). Specifically, we ran a one-way independent ANOVA to evaluate potential differences. Results indicate a non-significant main effect for attrition group on well-being scores, *F*(1, 59) = 0.01, *p* = 0.98, *_partial_η_p_^2^* < 0.01. Patterns suggest individuals who were invited but did not participate (*M* = 82.42, *SD* = 10.72) did not significantly differ on well-being scores compared to individuals who were invited and did participate (*M* = 82.31, *SD* = 12.19). Overall, while attrition can negatively affect the internal and external validity of the study (McBride, 2020), the identified null effect on well-being scores suggests the effect of attrition was minimal and does not largely impact the ability of the study to draw accurate conclusions.

To confirm individuals assigned to the control (*n* = 19) and CMA (*n* = 22) conditions were comparable in self-reported well-being scores, a one-way independent ANOVA was performed. Results revealed a non-significant main effect for condition on well-being scores, *F*(1, 39) = 1.23, *p* = 0.28, *_partial_η_p_^2^* = 0.03. This suggests control group participants (*M* = 80.05, *SD* = 13.56) reported similar well-being scores compared to CMA group participants (*M* = 84.27, *SD* = 10.82).

#### Exploratory factor analysis

To evaluate the factor structure of the MSIKPSM, we ran a principal component EFA using promax rotation. In addition, we specified some conditions to evaluate the factor analytic structure. Consistent with field recommendations, items were removed from inclusion in the final model if they generated a low communality score (<0.3; Costello and Osborne, 2005) or if corresponding factor loading coefficients were cross-loaded and/or small (<0.05; Stevens, 2012). Finally, factor loading coefficients less than 0.3 were suppressed.

Before running the EFA, the suitability of the data was inspected through the Kaiser Meyer–Olkin (KMO) and Bartlett’s Test of Sphericity statistics. The KMO value (0.68) exceeded the recommended value (0.6; Kaiser, 1960), and Bartlett’s Test of Sphericity (Bartlett, 1954) was significant, χ^2^(28) = 123.31, *p* < 0.01. In combination, these findings suggest the data were suitable to be analyzed via factor analytic procedures. The EFA revealed a three-factor solution. Eigenvalues for each of the three factors exceed the standard threshold (≥1) and, collectively, the three factors for accounted for 74.14% of the total variance. All item communalities were reported above the established threshold, and no cross-loadings were detected. Table 2 depicts the factor loadings of items. In general, items loaded onto the expected content themes: mental strength knowledge (two items), mental strength interest (two items), and mental strength perceived skills (four items). Internal consistency coefficients were slightly below expected standards for the knowledge (α = 0.66) and interest (α = 0.63) factor scores. However, the internal consistency coefficient for the perceived skills was good (α = 0.85).

**Table 2:** Factor loadings for the exploratory factor analysis (EFA) for the mental strength interest, knowledge, and skills assessment (MSIKSA).

	EFA loadings		
Items	Knowledge	Interest	Skills
How familiar are you with the concept of psychological or mental strengths?	0.99		
How familiar are you with your own specific psychological or mental strengths?	0.63		
How important is it for you to know your psychological or mental strengths well?		0.94	
How much interest do you have in learning more about your psychological or mental strengths?		0.76	
How easy is it for you to identify when you need to use your psychological or mental strengths?			0.96
How easy is it for you to access your psychological or mental strengths to complete your goals?			0.89
In general, how easy is it for you to access your psychological or mental strengths?			0.76
How easy is it for you to access your psychological or mental strengths to overcome adversity?			0.67

At first glance, it seems that lower coefficient scores (< 0.7) might reflect lower levels of reliability; however, these low scores are likely attributable to the small number of items in each factor domain (Cortina, 1993). In these cases, alpha should be considered the lower bound estimate of reliability and the number of items in each scale should be considered in making determinations about whether constructs with lower internal consistency coefficients can be used. Thus, we also calculated the inter-item correlation for each factor domain score. The item correlations were relatively high (*r* = 0.49 for knowledge and *r* = 0.46 for interest) in terms of effect size (Cohen, 1988), suggesting the items underneath each measure relate well to one another and are suitable for measuring the constructs at hand. As a result, we decided to retain these measures in the primary analysis.

### Primary findings

Means and standard deviations for each dependent variable across time and condition are presented in Table 3.

**Table 3:** The interaction effect between time and condition on mental strength-based indices.

	Time 1		Time 2		Time 3	
	Control (*n* = 19)	Intervention (*n* = 22)	Control (*n* = 19)	Intervention (*n* = 22)	Control (*n* = 15)	Intervention (*n* = 17)
Strength interest						
Mean	8.33	8.59	8.20	9.11	8.00	8.59
St. Dev.	1.98	1.37	1.78	1.31	2.00	1.28
*F*	0.08		2.34		1.01	
*p*	0.78		0.13		0.32	
*η^2^*	< 0.01		0.06		0.32	
Strength knowledge						
Mean	5.63	6.32	5.58	7.55	5.40	7.82
St. Dev.	1.42	1.32	1.35	1.47	1.50	1.24
*F*	2.56		19.69		25.04	
*p*	0.12		< 0.01		< 0.01	
*η^2^*	0.06		0.34		0.46	
Strength skill						
Mean	10.79	11.72	10.68	13.36	10.53	13.65
St. Dev.	2.82	2.35	2.92	2.65	3.46	2.78
*F*	1.35		9.48		7.95	
*p*	0.25		< 0.01		< 0.01	
*η^2^*	0.03		0.20		0.21	

#### Mental strength interest

**Figure 2:**
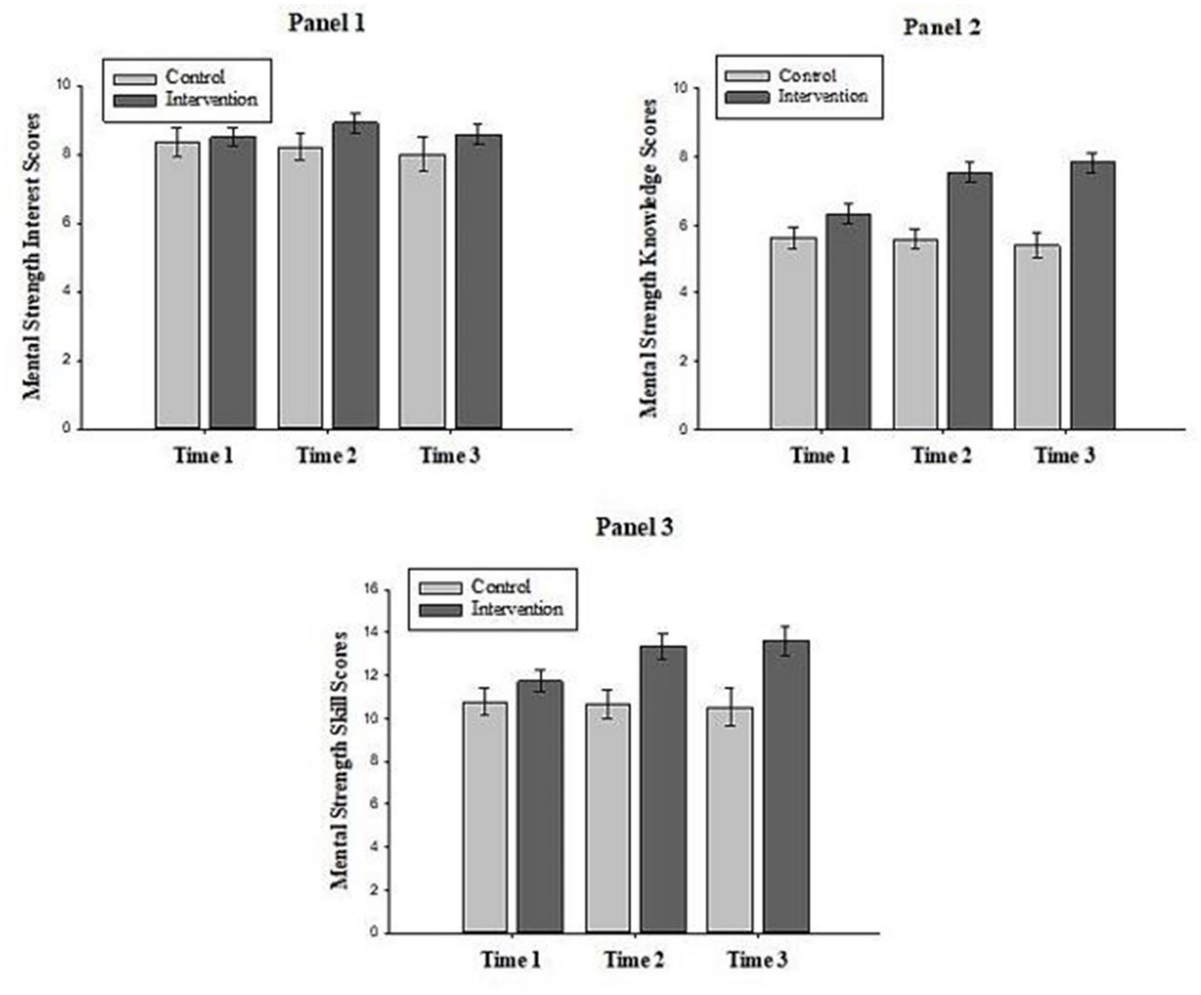
The interaction effect between time and condition on mental strength interest, knowledge, and skills. Error bars represent standard errors of the means.

As shown in Figure 2 (Panel 1), mean Mental Strength Interest (MSI) scores did not vary much across the three phases of study for participants in both conditions. These results were confirmed by the analysis, where a non-significant main effect for Time was revealed, *F*(2, 60) = 2.93, *p* = 0.11, *_partial_* η*_p_*^2^ = 0.07. Similarly, the effect of condition, *F*(1, 30) = 1.16, *p* = 0.29, and the interaction, *F*(2, 60) = 1.97, *p* = 0.15, were not significant. These findings indicate that, at each phase of the study, participants in the control condition reported comparable levels of MSI scores to participants in the CMA condition.

#### Mental strength knowledge

As shown in Figure 2 (Panel 2), reports of mental strength knowledge (MSK) did not differ between conditions for Time 1 but did differ between conditions at Time 2 and Time 3. These results were confirmed by the analysis, which revealed significant main effects for condition, *F*(1, 30) = 15.68, *p* < 0.01, *_partial_* η*_p_*^2^ = 0.34, and time, *F*(2, 60) = 6.03, *p* < 0.01, *_partial_* η*_p_*^2^ = 0.17, and a significant condition x time interaction effect, *F*(2, 60) = 16.86, *p* < 0.01, *_partial_* η*_p_*^2^ = 0.36. To isolate the source of the interaction, we conducted separate one-way ANOVAs for each time with condition as a factor. Consistent with expectations, reports of MSK at Time 1 were not significant, *F*(1, 39) = 2.56, *p* = 0.07. This suggests participants assigned to the control vs. CMA groups reported comparable MSK scores at baseline. However, there were reported condition differences at Time 2, *F*(1, 39) = 19.69, *p* < 0.01, *_partial_* η*_p_*^2^ = 0.34. Specifically, participants in the CMA condition (*M* = 7.55, *SD* = 1.47) reported higher MSK scores compared to participants in the control condition (*M* = 5.58, *SD* = 1.35). The size of this effect was large, *d* = 1.39 (Cohen, 1988). This pattern of scores highlights the beneficial effects of the CMA condition in terms of increasing MSK scores. Similarly, significant condition differences were detected at Time 3, *F*(1, 30) = 25.04, *p* < 0.01, *_partial_* η*_p_*^2^ = 0.46, where CMA participants (*M* = 7.82, *SD* = 1.24) reported higher scores on the MSK scale compared to control participants (*M* = 5.40, *SD* = 1.50). The size of this effect was large, *d* = 1.76. These findings indicate that the beneficial effects of the CMA condition on MSK scores persisted for 24 h after administration.

#### Mental strength skills

As shown in Figure 2 (Panel 3), mental strength skills (MSS) did not differ between conditions for Time 1 but did differ between conditions at Time 2 and Time 3. These results were confirmed by the analysis, which revealed significant main effects for condition, *F*(1, 30) = 5.87, *p* = 0.02, *_partial_* η*_p_*^2^ = 0.17, and time, *F*(2, 60) = 4.37, *p* = 0.02, *_partial_* η*_p_*^2^ = 0.13, and a significant condition x time interaction effect, *F*(2, 60) = 5.62, *p* < 0.01, *_partial_* η*_p_*^2^ = 0.16. To isolate the source of the interaction, we conducted separate one-way ANOVAs for each time with condition as a factor. Consistent with expectations, reported MSS at Time 1 were not significantly different by condition, *F*(1, 39) = 1.34, *p* = 0.25. This suggests that participants assigned to the control vs. CMA groups reported comparable MSS scores at baseline. However, there were condition differences at Time 2, *F*(1, 39) = 9.48, *p* < 0.01, *_partial_* η*_p_*^2^ = 0.20, with CMA participants (*M* = 13.36, *SD* = 2.65) reporting greater levels of MSS compared to control participants (*M* = 10.68, *SD* = 2.92). The size of this effect was large, *d* = 0.96. This pattern of scores highlights the beneficial effects of the CMA condition in terms of increasing MSS scores. Similarly, significant condition differences were detected at Time 3, *F*(1, 30) = 7.95, *p* < 0.01, *_partial_* η*_p_*^2^ = 0.21, where participants in the CMA condition (*M* = 13.65, *SD* = 2.78) reported higher ratings of MSS compared to participants in the control condition (*M* = 10.53, *SD* = 3.46). The size of this effect was large, *d* = 0.99. These findings indicate that the beneficial effects of the CMA condition on MSS scores persisted for 24 h after administration.

## Discussion

The present study is the first to evaluate the beneficial effects of a brief strength-based assessment protocol, rooted in CMA theory, on several strength-concordant outcomes (i.e., interest, knowledge, and perceived skill). The main findings suggest participants who completed the brief CMA protocol experienced large and practically meaningful increases in strength knowledge and perceived skill post-administration (Time 2) and 24 h after administration (Time 3) compared to control participants. However, these patterns of differences were not revealed for strength interest. Taken as a whole, these results are largely consistent with the prevailing CMA literature (Hanson and Poston, 2011; Durosini and Aschieri, 2021). Specifically, CMA procedures contribute to different facets of self-enhancement. However, these findings are somewhat unique compared to other experimental studies evaluating the benefits of CMAs. Our results suggest CMA procedures can be utilized in concert with positive psychological practices, offering new pathways for clinicians to approach strength-based assessment.

This study extends the function and utility of CMA employment. Notably, this experimental study was one of the first to develop and test the merits of strength-based assessment protocols rooted in CMA dynamics and processes. Consistent with similar CMA experimental studies (e.g., De Saeger et al., 2014), results offer preliminary evidence for the effectiveness of using CMA protocols with undergraduate students. Specifically, our brief assessment protocol increased reports of strength knowledge and perceived skill and these gains were maintained for 24 h post administration. Considering the paucity of research focused on strength-based CMAs, it is important to deconstruct our findings further by identifying the mechanisms directly responsible for the changes in participants’ scores. Two possible mechanisms warranting further consideration are the collaborative and individualized nature underlying our protocol. Qualitative reports associated with CMA experience highlight numerous benefits (e.g., increased efficacy, self-esteem, and empathy) respondents receive from collaborating with assessors in generating and discussing test feedback (Smith and Egan, 2017). Opportunities to find a sense of self-verification and validation, express and celebrate new perspectives, and generate new pathways to achieve well-being are just a few reported benefits associated with the presence of an assessor that potentially bolster respondents’ learning and exploration of their character strengths (Finn and Tonsager, 1997).

In addition, participants completing our brief assessment protocol may have benefited from the individualized components of the CMA protocol. Consistent with best CMA practices (Smith, 2016), participants were empowered to identify character strengths deemed most meaningful to their lived experience. Moreover, assessor-participant discussions built exclusively upon the participants’ definition of and relevant experiences with their chosen character strength. Such factors are therapeutically advantageous, providing respondents with more opportunities to integrate data-driven knowledge with their core values and identities (Aschieri et al., 2016; Smith, 2016). Future research should use experimental designs to evaluate whether the collaborative and individualized features significantly contribute to how individuals enhance their knowledge and use of character strengths.

The size of the main effects for condition on strength knowledge and perceived skill are also comparable, if not greater in strength, to those noted in studies investigating more quantitative methods of assessing and reviewing character strength (i.e., *VIA*-SI; e.g., Poston and Hanson, 2010; Forest et al., 2012). Evaluations such as these may pinpoint the unique therapeutic mechanisms inherent within CMAs associated with character strength. For instance, do the fundamental collaborative (e.g., interactions with assessor) and individualized (e.g., framing findings in the context of respondents’ lived experience) operations underlying our brief assessment protocol generate any additive benefits in helping respondents find meaning and grow within their strength knowledge and skill? Answers to questions like this may instruct mental health practitioners on how to use the most effective means of assessing character strength across different settings and circumstances.

Considering the nature and construction of our CMA protocol, our findings highlight the need for continued evaluation of the benefits of such an approach in multicultural spaces. As currently constructed, our CMA assessment procedure overlaps with two key cultural competencies. First, culturally responsive service is grounded in strength-based approaches, where professionals engage culturally diverse individuals, families, and communities in the acknowledgement of challenges as well as the promotion of resilience and well-being (American Psychological Association, 2017). Focused attention of culturally derived expressions of resilience and well-being increases opportunities to shed light on the full range of life experiences among non-WEIRD (Western, Educated, Industrialized, Rich, and Democratic; Hendriks et al., 2019) populations and minimize microinvalidations and minority stress (Vaughan et al., 2014). Second, qualitative, narrative, and contextual assessment approaches give voice and empowerment regarding how respondents frame, disseminate, and apply assessment findings in different cultural spaces (Laher and Cockcroft, 2017). Namely, qualitative procedures paint a more comprehensive picture of a respondent’s background and behavior, leading to more contextually bound and culturally responsive assessment findings. Considering our CMA approaches focus exclusively on strength, a key determinant of resilience and well-being, and provide ample opportunities for qualitative discourse, our protocol may offer unique opportunities to help respondents evaluate cultural identity knowledge and skills from a non-biased and balanced perspective. Moving forward, it is important for researchers to evaluate how our CMA framework bolsters cultural resources and promotes cultural identity development through experimental designs targeted to non-WEIRD populations.

Additionally, results did not reveal significant within-and between-participant effects on strength interest; control and CMA participants reported comparable strength interest scores across the duration of the study (Time 1, Time 2, and Time 3). These findings were somewhat surprising, as positive psychological theory suggests strength-based assessment procedures stimulate greater interest in using and developing character strengths (Owens et al., 2015). There are possible explanations for why no significant effects were detected. First, on average, participants in the control (*M* = 8.33) and CMA (*M* = 8.59) groups reported high levels of strength interest at baseline (Time 1), which may have limited our ability to detect meaningful changes in strength interest scores at Time 2 and Time 3. Second, it is possible that unaccounted cultural beliefs regarding the purpose and function of character strength limited our ability to detect changes in strength interest. Importantly, some participants may have been disinterested in pursuits designed to stimulate interest in character strength, preferring to pursue interests in activities that fix perceived deficits (Buckingham, 2007). Thus, the brevity of our assessment protocol (45 min) may have limited the influence or power needed to increase interest levels among participants who minimize the importance of character strength. Next, it is possible that self-selection (from Phase 1 to Phase 2) was responsible for the null effects. Participants who volunteered to participate in Phase 2 may be somehow different than individuals who chose not to volunteer. It is possible those who did not volunteer were less interested in the topics under investigation because they were less familiar or more skeptical about the potential benefits of participating in such activities. Paradoxically, these individuals might have benefited most, regarding increased interest, from engaging in the dynamic and idiosyncratic elements of the intervention. This pattern is consistent with the prevailing literature. Specifically, the advantages of employing certain positive psychological practices may be heightened for individuals with more reservations and fewer resources (e.g., low positive subjective experiences; Hurley and Kwon, 2013). Overall, it is important for future research to consider these methodological issues and cultural dynamics in re-evaluating the effects of different CMAs on strength interest scores.

Another unique feature of this study was the use of our strength interest, knowledge, and perceived strength scale (MSIKPSM). In designing the study, there was a shortage of psychometrically sound measures assessing these constructs. In response, we developed a brief measure assessing state-based fluctuations in participants’ strength interest, knowledge, and perceived strength. To ensure the measure demonstrated adequate properties for evaluation, we conducted an EFA. Results revealed a suitable three-factor solution, and the content of the items representing these factors corresponded well with strength interest, knowledge, and perceived skill themes, suggesting the measure demonstrates good content and basic factorial validity. Based on these findings, the MSIKPSM was appropriate to answer the questions posed in the study. Nevertheless, it is important to further evaluate the utility of this measure to ensure it is adequate to use in different settings and contexts across distinctive research designs. Importantly, future research needs to evaluate the structure of the measure using more sophisticated statistical procedures (i.e., confirmatory factor analysis). It is also important for researchers to evaluate internal and temporal consistency among the factor level items and convergent and predictive validity with theoretically salient measures of positive functioning with larger samples of participants.

Finally, our developed measure opens a larger discussion about the nature and measurement of strength-concordant outcomes. One of the more interesting aspects of the MSIKPSM is that only two items reflect content consistent with strength interest and knowledge subscales. At first blush, this aspect may be somewhat concerning as more items are better suited to increase reliability, offset random measurement error, and capture unique expressions of complex psychological constructs. Because of the smaller number of items in these subscales, researchers should remain skeptical regarding whether the MSIKPSM is a complete and accurate measure of strength interest and knowledge. Alternatively, it is possible that small item measures are entirely appropriate to assess these constructs. Notably, small item measures may be feasible for use when the construct under investigation is highly schematized for most individuals, unidimensional in nature, and largely reflects the subjective experience of a person (Robins et al., 2001). Currently, it is unknown whether strength interest and knowledge are uncomplicated, accessible, and organized constructs people can use across many settings. Moving forward, it is important for research to evaluate the nature and measurement of these constructs. Specifically, it is important researchers develop and validate a longer version of this scale. Once created, researchers then can take a comparison frame to determine if the longer versus shorter measures are highly correlated with each other and differentially correlated with theoretically relevant outcomes, including resilience, flourishing, and life satisfaction. Overall, the small number of items used to measure strength interest and knowledge is a potential concern yet sheds some light on important theoretical and measurement related topics worth exploring further.

Clinically, our findings suggest assessors can formulate their own strength-based evaluations through a multimethod (card sort and semi-structured interview) and qualitative procedure to increase respondents’ appreciation for character strength. Our brief assessment protocol offers a low-cost alternative to other standardized measures of strength-based assessment. Moreover, the magnitude of our findings speaks to the promise of employing card sorting procedures to evaluate character strength. Specifically, qualitative procedures are flexible in guiding participants’ exploration of different sets of character strength. In terms of implementation, we limited the structure of how participants explored their strength by having them choose and discuss the underlying nature of their top strength. However, our card sorting activity can easily be altered to meet the unique needs of different respondents. For instance, the card sorting process can be structured to help test-takers evaluate how top strengths help them marshal the energy needed to activate lower-level strengths, how top strengths are under-or overused, and how certain top strength combinations can bolster resilience. Overall, our results highlight some of the prevailing benefits of card sorting tasks; they offer a flexible, idiosyncratic, and dynamic method of introducing and discussing relevant wellness-oriented topics (Lenz and Roscoe, 2011).

While our findings are promising in diversifying how strengths are measured and evaluated at an intraindividual level, they do offer some limitations. Notably, the time investiture associated with our assessment process is extensive. Compared to other assessment methods using standardized self-report and interpretation platforms (e.g., *VIA*-SI Assessment System), our assessment process will not compete in terms of generating efficient findings for large scale projects. Instead, our assessment process seems more conducive for projects and services offered through more intimate or one-on-one mechanisms, like therapy, small group program work, or personal consultation/coaching.

Although our findings are promising, several limitations of the current study are acknowledged. First, the current study relied on self-report surveys to assess different strength-concordant outcomes. Self-report measures are subject to demand characteristics and social desirability concerns that may negatively affect the interpretation of our findings. To remedy these concerns, researchers are encouraged to re-evaluate the study’s questions using more behavioral or performance-based measures of strength interest, knowledge, and skill. Second, the sample consisted of college students, significantly limiting the generalizability of our findings. Similarly, the sample contained a disproportionate number of cisgender women compared to cisgender men. Although this gendered pattern is not uncommon for undergraduate samples (Klibert et al., 2021), evaluating how individuals with different gender identities benefit from a positive psychology-focused CMA is important in clarifying the utility and effectiveness of such an approach. Future studies should validate our findings with other groups of individuals, particularly individuals holding diverse gender identities (e.g., cisgender men, genderqueer, and non-binary) and clinical populations who have difficulty recognizing and accessing character strength. Third, although the current study attempted to determine how long positive gains are maintained (24 h), questions remain regarding whether our brief assessment protocol contributes to more permanent increases in the outcome variables. Evaluating participants’ responses after 24 h was a practical decision. In previous studies employing similar designs (Ford et al., 2017), attrition was a significant concern, especially with smaller *n* designs (Klibert et al., 2019). These concerns were exacerbated by the unpredictability of the COVID-19 pandemic, an emerging threat to data collection. Considering these concerns, we reduced the window of data collection as much as possible without sacrificing a longitudinal component, which seemed important given the criticisms of positive psychology research (van Zyl et al., 2023). We also believed this to be a reasonable plan of action, especially given the preliminary nature of the study. However, evaluating change scores across a compressed timeline may limit conclusions about the sustainability of the detected effects, another commonly critiqued issue in the positive psychology literature (van Zyl et al., 2023). Therefore, it is important for future research to utilize more complex longitudinal designs (e.g., to determine if participating in our brief assessment protocol maintains strength-based increases in strength knowledge and perceived skill over 2, 6, and 12 months). Fourth, it is unknown how the onset of the COVID-19 pandemic affected our findings. We prematurely terminated data collection in response to health and safety concerns, minimizing the sample size. This impacted the amount of power generated to detect significant effects. In addition, it is possible that anxiety stemming from early reports of a pandemic threat, as consumed through the media, served as a significant barrier to how participants identified and discussed characters strengths during the CMA protocol. It is important to re-evaluate the study’s questions post-pandemic to verify that our findings were not attributable to the psychological effects of living under threat of a health crisis. Finally, the higher levels of attrition noted from Phase 1 and Phase 2 were somewhat problematic. According to Schulz and Grimes (2002), researchers need to be concerned about bias when attrition rates supersede 20%. Our reported rate of attrition was 31.67%. Attrition can skew the results of a study making identified connections between independent and dependent variables suspect (Tierney and Stewart, 2004). However, we conducted some analyses to determine if attrition significantly affected our ability to detect meaningful findings and generalize those findings. Results of manipulation check analyses indicated the effects of attrition bias were minimal regarding the ranked covariate variable, well-being. While these results are encouraging and speak to the accuracy of our findings, they do not completely nullify the position that our findings are free from bias. Moving forward, it is important for future research to reanalyze our findings with studies protecting against high attrition.

In sum, our findings provide preliminary support for the use of a brief CMA protocol to increase strength knowledge and perceived skill and represent a significant extension to how character strengths are typically assessed and discussed (Klibert and Allen, 2019; Ruch et al., 2020). Moving forward, it is important to determine whether our brief assessment protocol, which is more qualitative in design, is more effective in eliciting increases in different strength-concordant outcomes compared to more traditional and standardized assessment protocols. Additionally, future research is needed to validate the efficacy of using such an approach to increase strength-concordant outcomes for longer periods of time and with diverse samples. Nonetheless, these results offer a promising pathway for clinicians to assess different features associated with character strength.

## Data availability statement

The raw data supporting the conclusions of this article will be made available by the authors, without undue reservation.

## Ethics statement

The studies involving human participants were reviewed and approved by Georgia Southern IRB; Study H19469. The patients/participants provided their written informed consent to participate in this study.

## Author contributions

JK was responsible for designing the project, collecting data, and writing up the largest portion of the manuscript. MS was responsible for collecting the data and writing up significant portions of the manuscript. BW was responsible for designing the study, writing up small sections of the manuscript, and editing the manuscript. CY was responsible for writing up small sections of the manuscript and general manuscript editing. CP was responsible for writing up small sections of the manuscript and targeted editing. HH was responsible for writing up small sections of the manuscript. AL was responsible for writing up small sections of the manuscript and targeted editing. Finally, HS directly contributed to the study through the implementation of the Rank Set Sampling techniques as well as writing up this section of the manuscript. All authors contributed to the article and approved the submitted version.

## Conflict of interest

The authors declare that the research was conducted in the absence of any commercial or financial relationships that could be construed as a potential conflict of interest.

## Publisher’s note

All claims expressed in this article are solely those of the authors and do not necessarily represent those of their affiliated organizations, or those of the publisher, the editors and the reviewers. Any product that may be evaluated in this article, or claim that may be made by its manufacturer, is not guaranteed or endorsed by the publisher.

## Footnotes

^1^ https://www.sona-systems.com/
